# Supplementary material for: Modulation of In Vitro Macrophage Responses via Primary and Secondary Bile Acids in Dogs
Source: Animals (Basel). 2023 Nov 30;13(23):3714. doi: 10.3390/ani13233714 (PMC10705343; doi:10.3390/ani13233714)
Supplement: Supplementary file 1 [file animals-13-03714-s001.zip › animals-2697836-supplementary.pdf]

Supplementary Material

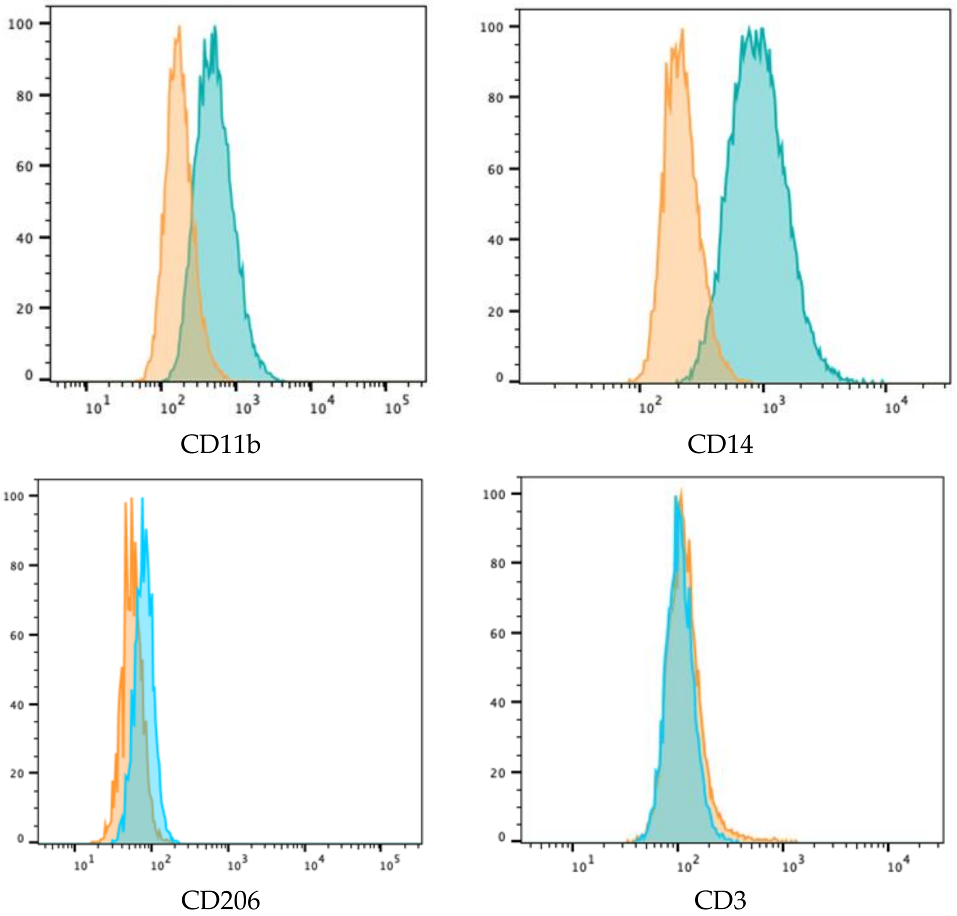

Figure S1. Results of single-color flow cytometric analysis of canine MH588 cells. Depicted are isotype controls (orange) and specific marker staining, with counts normalized to mode. Antibodies utilized were CD11b (rat anti-mouse conjugated phycoerythrin; Invitrogen), CD14 (mouse anti-human conjugated pacific blue; BioRad), CD206 (mouse anti-human phycoerythrin; Beckman Coulter), CD3 (mouse anti-dog conjugated fluorescein; BioRad).

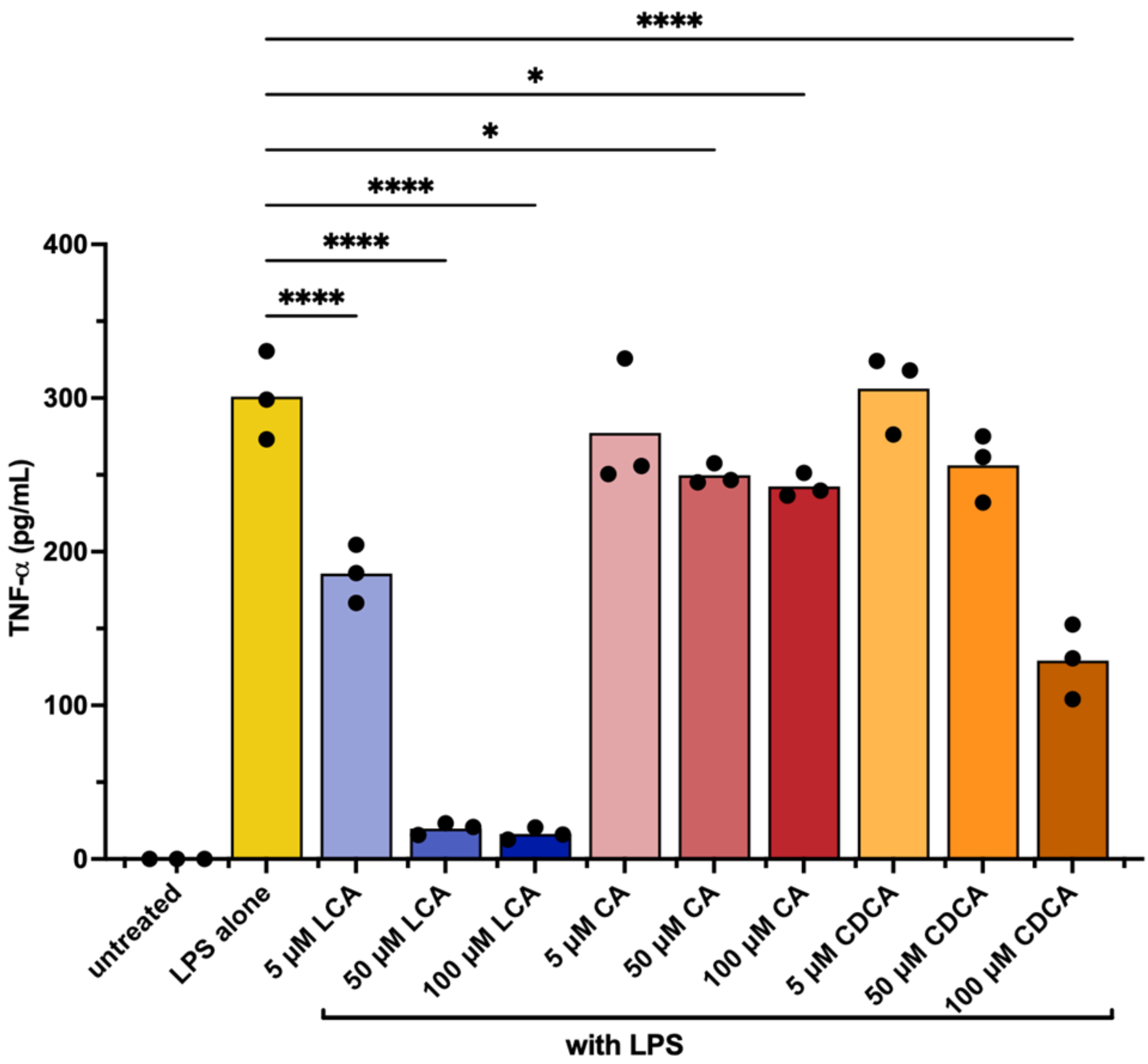

Figure S2. TNF-⊚ concentrations in supernatants from canine macrophages (MH588) treated with LPS alone, or with bile acid at 25 µM, incubated 2 hours, then stimulated with LPS at 300 ng/mL. Cell culture supernatants harvested 48 hours later and TNF-a concentration determined via ELISA. Values were compared with one-way ANOVA with the Holm-Sidak multiple comparison test. Horizontal bars with asterisks indicate significantly different mean values compared to control treatment of LPS alone; * $p < .05$, **** $p < .0001$.

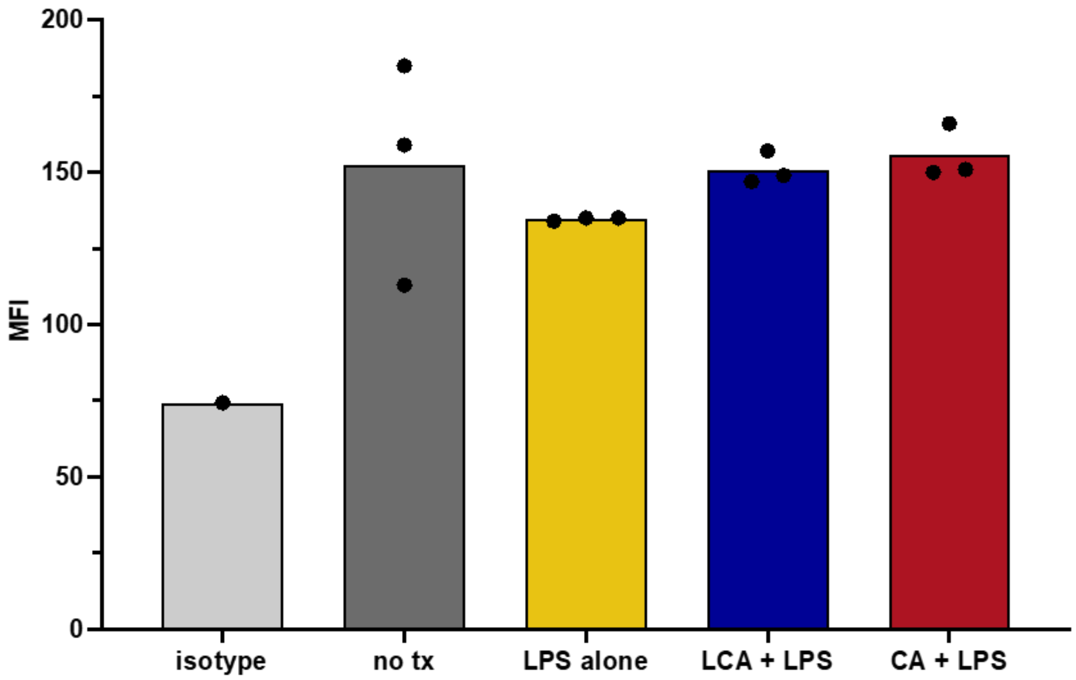

Figure S3. Mean fluorescence intensity (MFI) for TGR5 in canine macrophages (MH588) after 48 hours after treatment with no additives, LPS alone, or bile acid at 25 µM followed 2 hours later by stimulation with LPS at 100 ng/mL. Cells immunostained with unconjugated anti-TGR5 antibody followed by cyanine3 (Cy3) conjugated secondary.

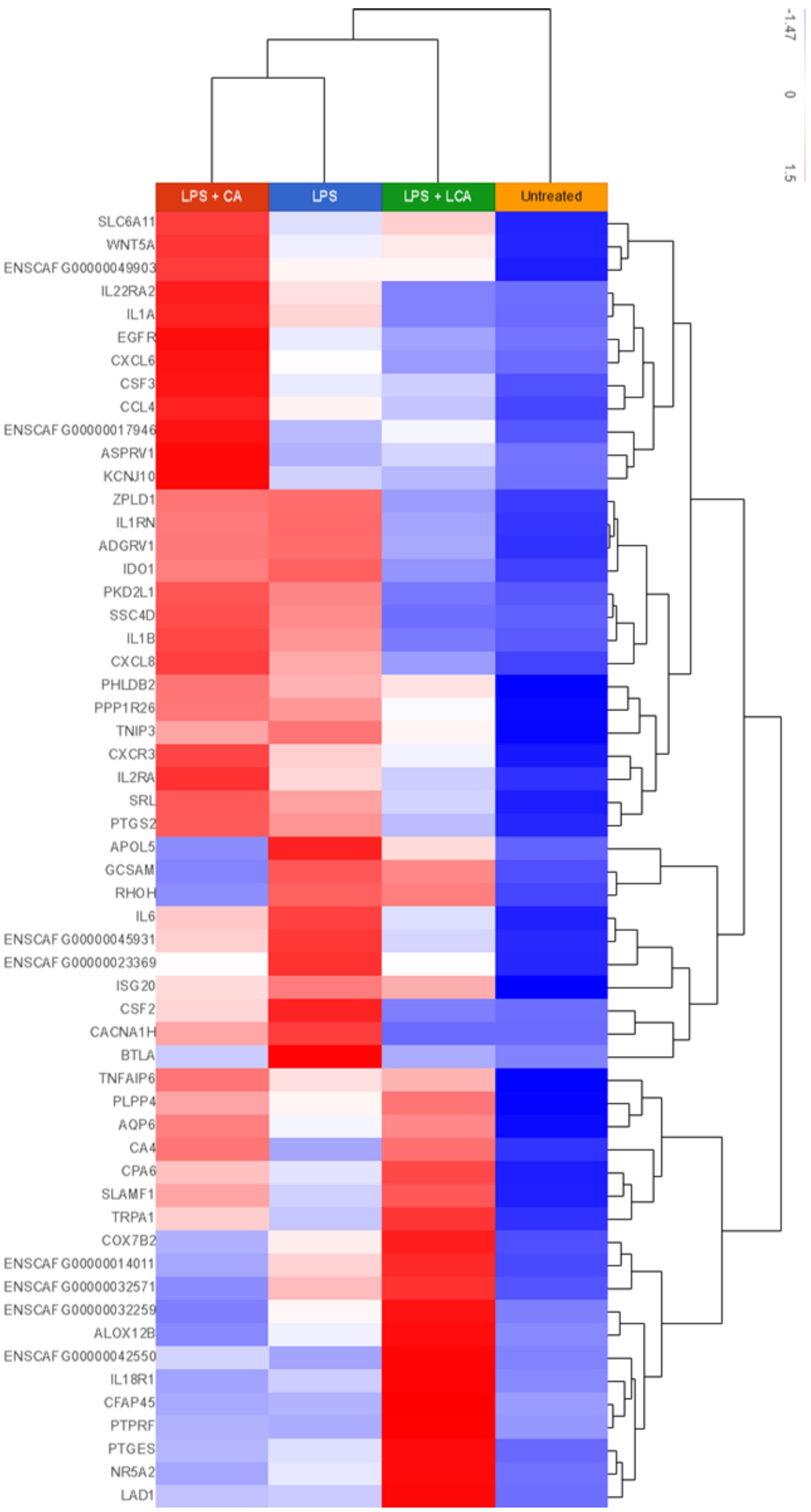

Figure S4. Heatmap depicting the impact of LPS stimulation on gene expression in canine macrophages (MH588 cells) with or without CA and LCA pre-treatment compared to untreated cells based on gene-specific analysis (GSA). Red boxes indicate higher relative expression and blue boxes represent lower relative expression.

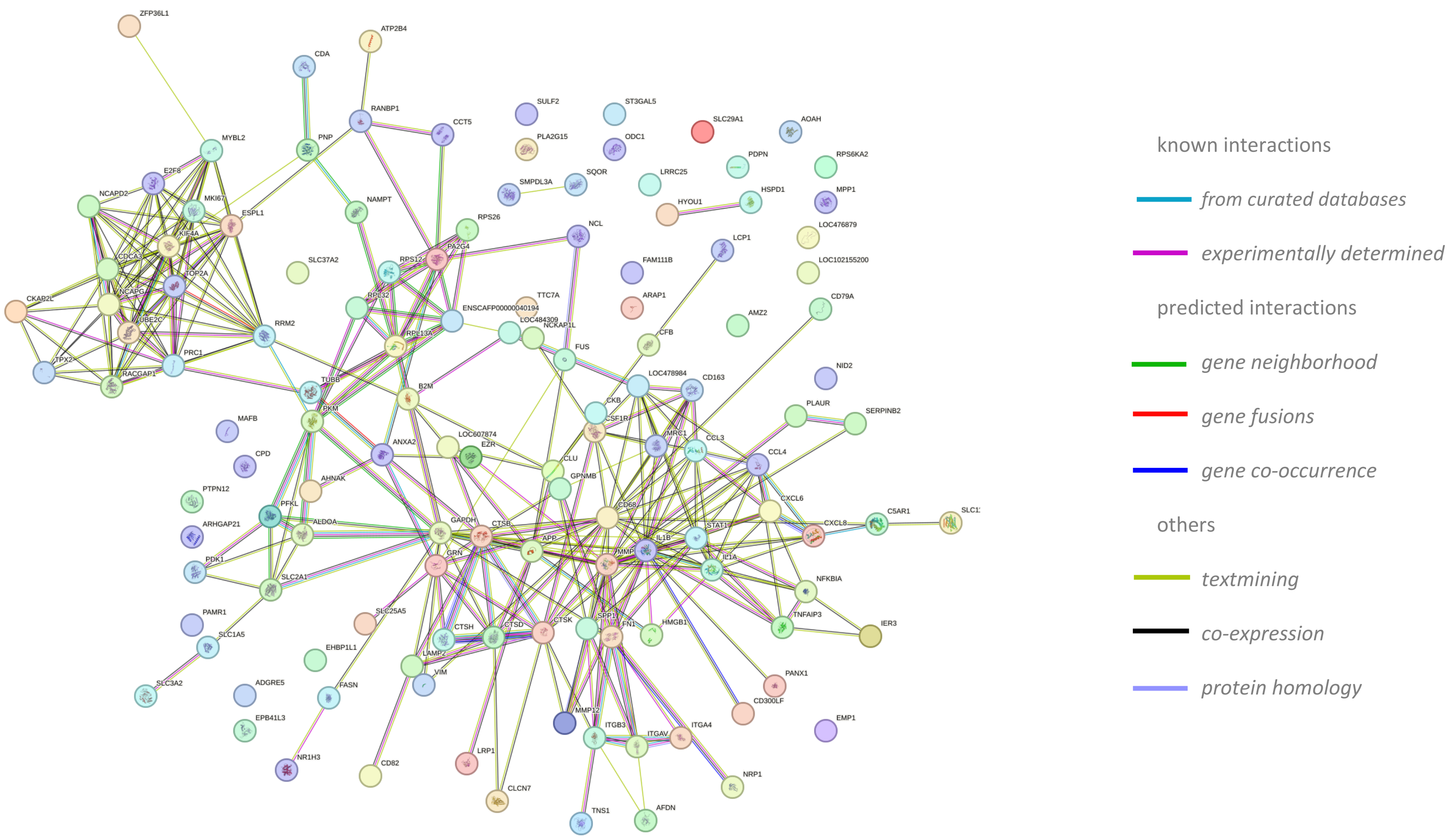

Figure S5. Protein network interaction prediction based on 130 input genes exhibiting similar change in expression after treatment with CA or LCA and LPS sitmulation. Lines connecting protein nodes indicate both functional and physical protein associations, with color representing basis of interaction evidence. Network developed with no maximum number of interactors and with a minimum required interaction score of 0.
